# Karyotype–phenotype associations in turner syndrome: a multicenter retrospective cohort study

**DOI:** 10.3389/fendo.2026.1830126

**Published:** 2026-07-07

**Authors:** Rama Watad, Sara Al Jneibi, Sareea Al Remeithi, Rasha Hassan Beck, Noura Al Hassani, Asma Deeb

**Affiliations:** 1 Paediatric Endocrine Division, Sheikh Shakhbout Medical City, Abu Dhabi, United Arab Emirates; 2 Paediatric Endocrine Division, Sheikh Khalifa Medical City, Abu Dhabi, United Arab Emirates; 3 Gulf Medical University, Ajman, United Arab Emirates; 4 Paediatric Endocrine Division, Tawam Hospital, Al Ain, United Arab Emirates; 5 Faculty of Medicine and Health Science, United Arab Emirates (UAE) University, Al Ain, United Arab Emirates; 6 Faculty of Health & Science, Khalifa University, Abu Dhabi, United Arab Emirates

**Keywords:** growth hormone, karyotype, menarche, puberty, Turner syndrome

## Abstract

**Background:**

Establishing clear links between different Turner syndrome (TS) karyotypes and phenotypes would help inform discussions around likely disease trajectories. Here we explored associations between karyotype, clinical features, and growth hormone (GH) responses in individuals with TS.

**Methods:**

This was a retrospective observational study of 86 patients diagnosed with TS attending three endocrine centers in Abu Dhabi, UAE. Clinical and genetic data were retrieved from the medical records. Changes in height standard deviation score (SDS) over time were assessed with repeated measured ANOVA with Tukey’s *post hoc* test.

**Results:**

The median (IQR) age of participants at diagnosis was 9.3 (6.5, 13.4) years. 44.2% were classical monosomies, 18.6% were mosaic, and 37.2% were structural X-chromosome abnormalities (isochromosome Xq, deletions, ring chromosome). GH treatment was started at a mean (SD) of 8.7 (4.0) years, which resulted in a significant increase in mean (SD) height SDS, with a greater increase in the first year [0.59 (0.13)] than between years 1 and 3 [0.22 (0.13)]. Spontaneous menarche occurred in 1/23 (4.3%) of evaluable 45, X individuals, compared with 6/12 (50.0%) of mosaic and 10/22 (45.5%) of structural X-chromosome abnormality cases (p=0.002), and autoimmune hypothyroidism was significantly more common in individuals with structural X-chromosome abnormalities (15/32, 46.9%, vs 5/37, 13.5%, and 1/16, 6.2%, in monosomy and mosaic cases, respectively; p<0.001; adjusted OR 5.22 [95% CI 1.55–17.53] after adjustment for current age). No detectable association was observed between karyotype and one-year GH response in the available analyses.

**Conclusions:**

TS remains challenging to recognize and manage, and early detection and management are crucial to optimize growth, pubertal development, and quality of life. Knowledge of karyotype-phenotype associations helps to manage patient and family expectations and plan likely future management.

## 1 Introduction

Turner syndrome (TS) is one of the most common sex chromosome disorders, with an incidence of 25–50 per 100, 000 females (1, 2). TS is caused by either lack or non-function (through structural abnormalities) of the second X chromosome, with or without mosaicism (1, 2). The major genetic abnormalities causing TS include 45, X monosomy and various forms of mosaicism such as 45, X/46, XX and 45, X/46, XY, while structural X-chromosome abnormalities include isochromosome Xq, ring X, and partial Xp and Xq deletions (3). Although traditionally described as a presence of physical features such as characteristic facial features, webbing of the neck, and peripheral lymphedema (4), TS is now best viewed as a multisystem disorder associated with a constellation of features including short stature, pubertal delay, and neurological, cardiovascular, renal, skeletal, and endocrine/metabolic abnormalities (1, 2). Although often diagnosed antenatally or in childhood when girls exhibit linear growth failure or primary amenorrhea, the clinical heterogeneity of TS means that diagnostic opportunities are often missed, delaying access to the optimal multidisciplinary care that is then required throughout life to manage the multisystem comorbidities that characterize the syndrome.

Early diagnosis and treatment of TS are therefore essential (1, 5), and establishing clear links between different karyotypes and phenotypes would be helpful to inform discussions around likely disease trajectories, helping patients and families to prepare for future medical events and anticipating psychological impacts associated with the condition, such as through loss of fertility, sexual development, and general health (6). However, while it is generally considered that the severity of clinical features in TS tends to correspond to the amount of X chromosome loss (7), with the monosomy 45, X karyotype the most severe, there are conflicting results on karyotype-phenotype associations (8), and relationships between karyotypes and clinical features and outcomes remain uncertain (1, 5). There is still a need for data to support aggregate analyses to firmly establish karyotype-phenotype correlations to inform clinical decision-making.

We therefore conducted a retrospective cohort analysis of all patients diagnosed with TS who attended three tertiary healthcare centers in the United Arab Emirates between June 2023 and May 2024. This analysis aimed to systematically evaluate the relationships between karyotype variations, clinical phenotypic features, and responses to growth hormone (GH) therapy. In doing so, we provide further evidence supporting specific karyotype-phenotype associations to help practical discussions with patients and families, as well as clarifying the impact of karyotype on growth hormone responses.

## 2 Methods

### 2.1 Study design and ethics

This was a retrospective observational study of 86 patients diagnosed with Turner syndrome attending three endocrine centers in Abu Dhabi, UAE: Sheikh Shakhbout Medical City (SSMC), Sheikh Khalifa Medical City (SKMC), and Tawam Hospital between June 2023 and May 2024. The study is reported according to the STROBE framework (9). This study protocol was reviewed and approved by the Institutional Review Board of the SSMC, approval number 22.07.2019 [RS-600].

Clinical and genetic data were retrieved from the medical records and input into a structured database to record clinical parameters: age at diagnosis, pubertal history including first follicle-stimulating hormone (FSH) level and details of induction therapy, associated comorbidities, growth hormone (GH) treatment details, and height SDS recorded at the start of treatment and after one and three years of treatment. Autoimmune thyroid disease included autoimmune hypothyroidism confirmed by thyroid autoantibody positivity.

### 2.2 Karyotyping

Karyotyping was performed in one centralized cytogenetics laboratory following International System for Human Cytogenomic Nomenclature (ISCN) conventions, with metaphase cell counts shown in parentheses in **Supplementary Table S1** where documented and typically following the current best-practice minimum of 30 metaphases per first-line test (1). Molecular-level analyses (fluorescence *in situ* hybridization, chromosomal microarray, or exome/genome sequencing) were not systematically performed.

### 2.3 Phenotype descriptors

For phenotype analyses, karyotypes were grouped into three categories: (1) classical monosomy (pure 45, X with no second cell line detected); (2) mosaic karyotypes (45, X with one or more additional cell lines containing a structurally intact second sex chromosome: 45, X/46, XX, 45, X/47, XXX, and 45, X/46, XY); and (3) structural X-chromosome abnormalities, comprising isochromosome Xq [i(X)(q10) and idic(X)(p11.2)], partial deletions of Xp or Xq, and ring X chromosomes [r(X)]. The third category is cytogenetically heterogeneous and was treated as a single group to permit between-group comparisons given the cohort size, and the breakdown of individual cytogenetic subtypes is shown in **Supplementary Table S1**.

### 2.4 Statistical analysis

Data normality was assessed with the Shapiro-Wilk test. Parametric continuous data are presented as mean (SD), non-parametric continuous data as median (interquartile range, IQR), and categorical data as counts (%). Changes in height SDS over time were assessed with repeated measures ANOVA with Tukey’s *post hoc* test. Associations between karyotype and clinical characteristics were tested with Welch’s ANOVA or chi-squared tests. Where the number of events per variable in logistic regression or the number of observations per predictor in linear regression exceeded the conventional minimum of 10 (10), multivariable models were fitted to assess whether the univariable karyotype-phenotype associations persisted after adjustment for the principal confounders of current age for the autoimmune hypothyroidism analysis (logistic regression), and GH dose, baseline height SDS, and age at GH initiation for the one-year growth response analysis (linear regression). The spontaneous menarche analysis (17 events) and the three-year growth response analysis (observations-per-predictor 9.6) did not meet this threshold and are reported as univariable analyses. All statistical analysis was conducted in Jamovi v.2.6.44.0 or with Python v3.13 with the *matplotlib* and *seaborn* packages.

## 3 Results

### 3.1 Cohort characteristics and karyotype distribution

**Figure 1:**
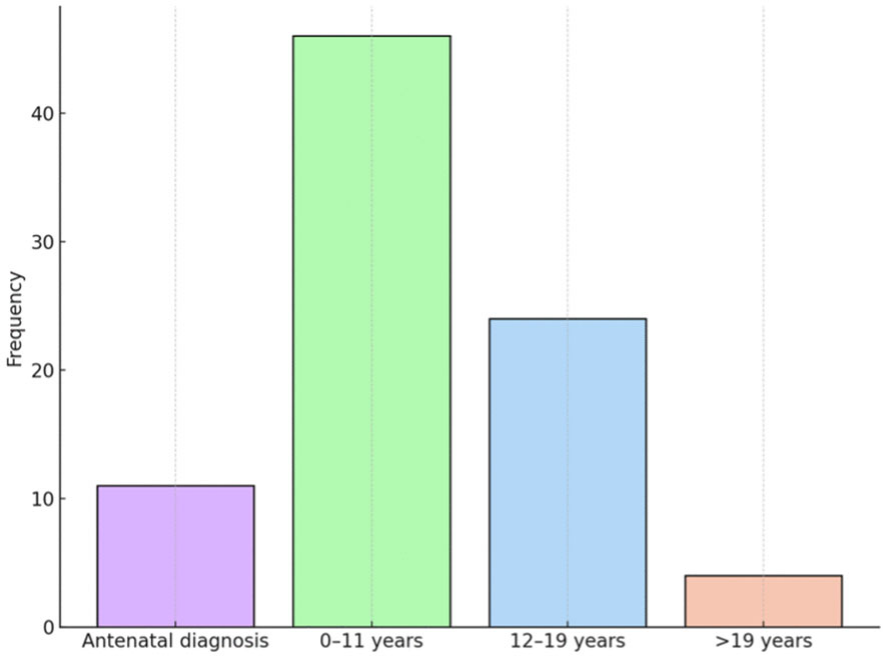
Age distribution of the study cohort.

The demographics, reason for karyotyping, and overall karyotype distribution are presented in **Table 1**. The median (IQR) age of the study population at diagnosis, excluding individuals diagnosed antenatally, was 9.3 (6.5, 13.4) years (n=75). 46/86 (53.5%) were diagnosed in the 0–11-year age group, 29.1% (25/86) in adolescence, and 4.7% (4/86) as adults (**Figure 1**).

**Table 1:** Clinical and genetic characteristics of the study cohort.

Variable	n (%) or Median (IQR)
Age at diagnosis (excluding antenatal diagnoses), years	
Median (IQR)	9.3 (6.5, 13.4)
Range	1.3 - 31.4
Height SDS at diagnosis	-3.0 (-3.8, 2.4)
Median (IQR)	
Reason for karyotyping	
Ambiguous genitalia	1 (1.2%)
Antenatal diagnosis	11 (12.8%)
Coarctation of Aorta	1 (1.2%)
Confirmed with cardiac surgery	1 (1.2%)
Dysmorphic features, cardiac anomalies	1 (1.2%)
Lymphedema	3 (3.5%)
Oligomenorrhea	1 (1.2%)
Primary amenorrhea	6 (7.0%)
Primary amenorrhea and short stature	7 (8.1%)
Secondary amenorrhea	1 (1.2%)
Short stature	51 (59.3%)
Short stature and celiac disease	1 (1.2%)
Short stature and hypothyroidism	1 (1.2%)
Karyotype subtype	
Deletion	8 (9.3%)
Isochromosome	20 (23.3%)
Monosomy	38 (44.2%)
Mosaicism with triple X	3 (3.5%)
Mosaicism	11 (12.8%)
Ring chromosome	4 (4.7%)
Y mosaicism	2 (2.3%)
Simplified karyotype	
Monosomy	38 (44.2%)
Mosaicism	16 (18.6%)
Structural X-chromosome abnormalities	32 (37.2%)
Number of systemic comorbidities	
Median (IQR)	2 (1, 4)
Range	0-8
Systemic comorbidities	
Autoimmune hypothyroidism	21 (25%)
Subclinical hypothyroidism	8 (9.4%)
Hyperthyroidism	0 (0.0%)
Celiac disease	10 (16%)
Type 1 diabetes	1 (1.2%)
Type 2 diabetes	5 (5.8%)
Congenital heart disease	36 (42%)
Renal anomalies	16 (20%)
Hypertension	9 (10%)
Dyslipidemia	18 (33%)
Musculoskeletal abnormalities	19 (22%)
Conductive hearing loss	14 (16%)
Sensorineural hearing loss	2 (2.3%)
Recurrent otitis media	20 (23%)
Skin disorders	20 (23%)
Eye disorders	19 (22%)

The majority of individuals were referred for karyotyping due to short stature (60/86, 69.8%; with or without other conditions) or menstrual disorders (15/86, 17.4%); 11/86 (12.8%) were diagnosed antenatally. Among the 11 antenatally diagnosed cases, 9 had a 45, X karyotype and 2 had 45, X/46, XX mosaicism; the specific antenatal indication (e.g., increased nuchal translucency, abnormal non-invasive prenatal testing, structural anomalies on ultrasound, or routine screening) was not consistently documented in the clinical records. Reflecting the frequency of short stature, the median (IQR) height SDS at diagnosis was -3.0 (-3.8, 2.4).

The full list of karyotypes detected in the population is shown in **Supplementary Table S1**, and the subtype distribution is shown in **Table 1**. Nearly half (38/86, 44.2%) were classical monosomies (45, X), while 20/86 (23.3%) were isochromosome Xq structural variants, 8/86 (9.3%) were deletion variants, 4/86 (4.7%) were ring chromosomes, and 16/86 (18.6%) were mosaicisms (including Y mosaicism and with triple X).

### 3.2 Comorbidities

Individuals with TS had a median (IQR) of 2 (1, 4) associated systemic comorbidities: 30/86 (34.4%) had confirmed thyroid abnormalities (autoimmune and subclinical hypothyroidism), 36/86 (42%) had congenital heart disease (CHD), while 16/86 (20%) had renal anomalies, especially horseshoe kidney (8/16). Among the 36 CHD cases, the specific lesions were bicuspid aortic valve alone in 11 (30.6%), coarctation of aorta alone in 3 (8.3%), bicuspid aortic valve with coarctation in 6 (16.7%), aortic regurgitation/dilatation in 1 (2.8%), and other unspecified defects in 15 (41.7%); left-sided obstructive lesions thus accounted for 20/36 (55.6%) of CHD cases. Musculoskeletal abnormalities (19/86, 22.1%; especially flat feet), recurrent otitis media (20/86, 23.3%), dermatological conditions (20/86, 23.3%; especially atopic dermatitis/eczema and alopecia), and ophthalmological conditions (19/86, 22.1%; especially visual acuity problems) were each present in around one-fifth of the cohort.

### 3.3 Pubertal development

Of 57 individuals evaluable for menarche status, 17 (29.8%) had attained spontaneous menarche, at a mean (SD) age of 13.2 (1.38) years (**Table 2**). Thirty-five individuals required induction of puberty as per hospital protocols and physician experience, and consistent with hypogonadism, the mean (SD) FSH level in these individuals was 57.3 (32.7) mIU/ml (normal ranges: before puberty: 0 to 4.0 mIU/ml, during puberty: 0.3 to 10.0 mIU/ml).

**Table 2:** Puberty characteristics of the study population.

Characteristic	n = 86
Age of spontaneous menarche, years [mean (SD)]	13.2 (1.38)
Range	11-16
Spontaneous menarche	
No	40 (47%)
Yes	17 (20%)
N/A	29 (34%)
Puberty induction	
No	20 (24%)
Yes	35 (41%)
N/A	31 (35%)
Age at puberty induction, years [median (IQR)]	13.4 (12.0, 14.8)
First FSH level (mIU/ml) [median (IQR)]	57.3 (32.7)

### 3.4 Growth hormone therapy and height trajectories

**Figure 2:**
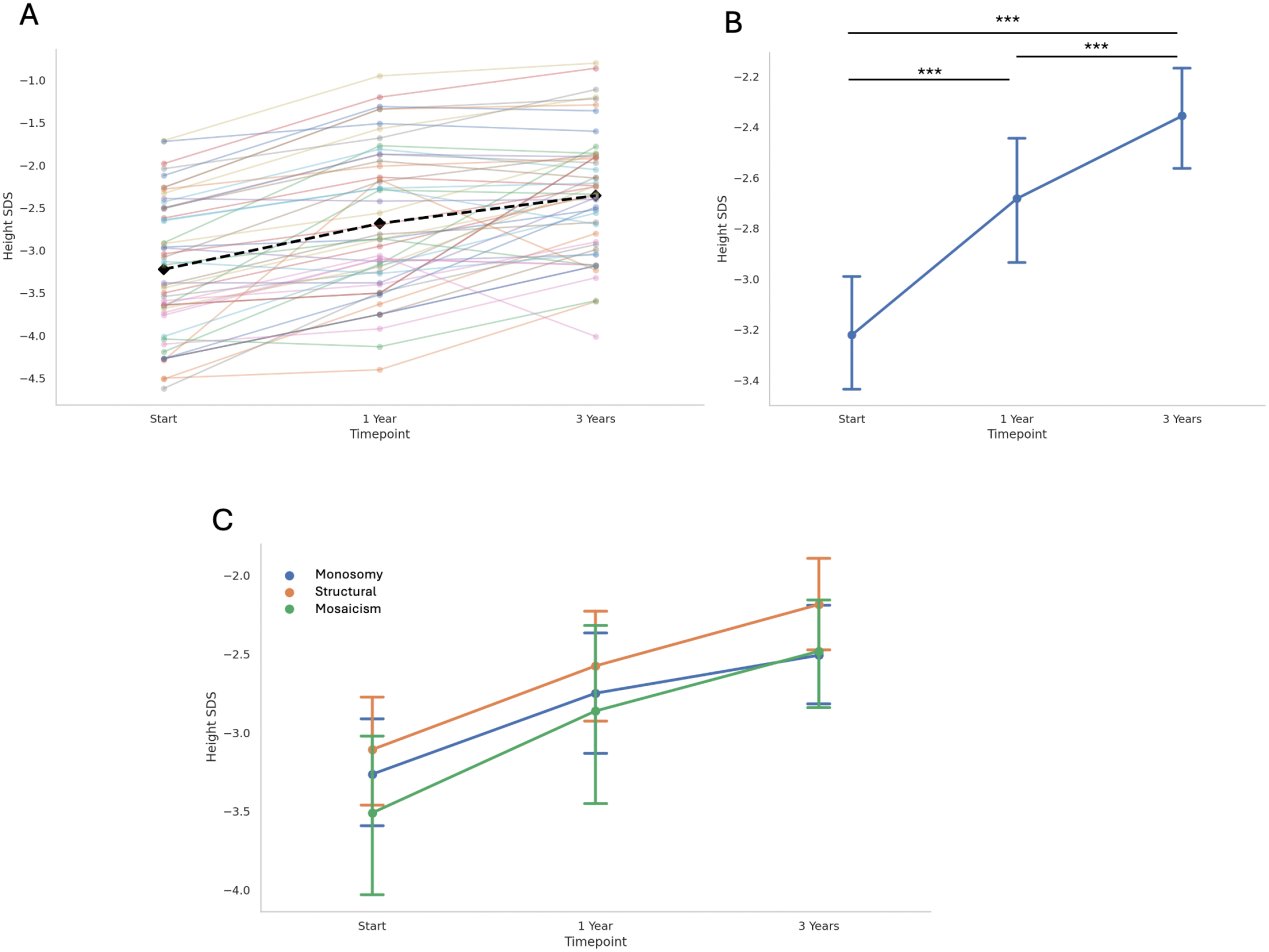
**(A)** Individual patient and **(B)** group trajectories in height SDS after starting growth hormone treatment. **(C)** Height SDS trajectories according to karyotype. ***p<0.001.

Sixty-nine individuals received GH treatment at a mean (SD) dose of 0.05 (0.04) mg/kg started at a median (IQR) of 9.0 (5.1, 11.7) years of age. Height was measured at the start of treatment and at one and three years after starting treatment. Individual patient height SDS trajectories showed that 45/50 (90%) individuals showed a consistent upward trend in height SDS over the treatment period (**Figure 2A**). Mean (SD) height SDS gain during the first year after starting GH treatment (**Figure 2B**) was higher in the first year [0.59 (0.13)] compared with between years 1 and 3 [0.22 (0.13)], although 3-year SDS values [-2.4 (0.7)] remained well below the population average. Repeated measures ANOVA (p<0.001) with Tukey’s *post hoc* test (p<0.001 for difference between height at start of treatment and 1 and 3 years, respectively) confirmed the changes over time. There was no correlation between % growth change between baseline and 1 and 3 years and GH dose (r=0.23 and r=0.07, respectively; p>0.05) or age at GH initiation (r=-0.1 and r=-0.01, respectively; p>0.05).

### 3.5 Karyotype–phenotype associations

Finally, we examined whether karyotype was associated with any clinical characteristics (age, FSH, age at menarche, height SDS, height SDS changes on treatment, and systemic conditions), grouping the karyotype into classical monosomies, mosaicism, and structural X-chromosome abnormalities (isochromosome Xq, deletions, ring chromosome). Univariable associations are shown in **Supplementary Tables S2**, **S3**. No associations were observed between karyotype group and age at diagnosis, height SDS at diagnosis, or growth response to GH therapy (all p>0.05). Spontaneous menarche occurred in 1/23 (4.3%) evaluable individuals with a 45, X karyotype, compared with 6/12 (50.0%) individuals with mosaicism and 10/22 (45.5%) of those with structural X-chromosome abnormalities (chi-squared p=0.002). Conversely, induction of puberty was required in 21/22 (95.5%) evaluable monosomy cases, compared with 4/11 (36.4%) and 10/22 (45.5%) of mosaicism and structural cases, respectively. Autoimmune hypothyroidism was significantly more common in individuals with structural X-chromosome abnormalities (46.9% vs 13.5% in monosomy cases and 6.2% in mosaic cases; chi-squared test p<0.001), including for deletion, isochromosome Xq, and ring chromosome cases (**Supplementary Table S4**). No association between karyotype and GH response was detected (**Figure 2C**).

Where the data permitted, we performed multivariable analyses (**Supplementary Table S5**). In multivariable logistic regression for autoimmune hypothyroidism (n=85; karyotype + current age), the structural-vs-monosomy association remained significant (adjusted OR 5.22, 95% CI 1.55–17.53, p=0.008), with current age also independently associated with autoimmune hypothyroidism (adjusted OR 1.07 per year, 95% CI 1.01–1.14, p=0.025). A sensitivity analysis of structural-vs-non-structural karyotypes (events-per-variable 10.5) gave a consistent estimate (adjusted OR 7.29, 95% CI 2.32–22.97, p=0.001). In multivariable linear regression for the one-year change in height SDS (n=58; karyotype + GH dose + baseline SDS + age at GH initiation), no covariate was significant (overall F p=0.10), consistent with the univariable findings.

## 4 Discussion

Here we present the clinical, genetic, and treatment response characteristics of a multi-center cohort of individuals with TS, seeking to explore and clarify karyotype-phenotype associations. Reflecting the considerable phenotypic heterogeneity of TS (1), a third of diagnoses were in adolescence or adulthood. Although heterogeneous, our cohort presented with features typically described for TS including short stature, ovarian insufficiency, and a range of neurological, cardiac, renal, and skeletal comorbidities. The spectrum of karyotypes represented in the cohort allowed for exploration of karyotype-phenotype associations. Consistent with previous reports, we observed more severely delayed puberty in individuals with a 45, X karyotype and more frequent autoimmune hypothyroidism in individuals with structural X-chromosome abnormalities (deletions, isochromosome Xq, and ring chromosome). Individual and group trajectories showed the expected positive impact of GH therapy on growth, with no detectable difference in response between karyotype groups.

Despite wide recognition of the condition, TS still represents a diagnostic challenge due to its clinical heterogeneity (1), and no individual sign or symptom, even short stature, is ubiquitous in girls and women with TS. Reflecting this, diagnoses were made across the life spectrum in our cohort: about one in eight were antenatal diagnoses and about half of diagnoses were in the 0–11-year age group (usually due to short stature), 29.1% in adolescence (usually due to pubertal delay), and a few as adults prompted by menstrual irregularities. In that regard, our cohort was representative of the usual clinical spectrum of TS (1, 5). Recent clinical practice guidelines on the management of girls and women with TS emphasize the importance of an early diagnosis (1), as achieving this allows for early commencement of GH therapy when growth failure is at its greatest, thereby preventing loss of height potential (11). We achieved commencement of GH therapy before six years of age in 22/69 individuals treated with GH (31.9%), with five girls treated before three years of age, in line with recommendations that GH is indicated in very young children (from 2 years) with TS with evidence of growth failure or short stature (1).

As expected, GH therapy had a positive impact on growth, with significant individual and group increases in height SDS over three years. Treatment responses were greater in the first year than later, mirroring previous findings in a cohort of 194 individuals with TS, who showed very similar start (~-3.0) and final (~-2.0) SDS height values after three years of treatment and similar growth trajectory with early enhanced response (12). Other studies have similarly reported accelerated responses in year one, which tailed off after (13, 14). We did not detect any correlations between growth response and GH dose or age of starting therapy, as noted in other studies (11, 15). In a multivariable model for the one-year response adjusting for karyotype, GH dose, baseline height SDS, and age at initiation, no covariate was significant (**Supplementary Table S5**), consistent with the univariable findings. Bone age was not systematically recorded across the three participating centers, precluding adjustment for skeletal maturation, and the three-year follow-up does not extend to final adult height or long-term variability in treatment response. Nevertheless, an early diagnosis remains a clinical imperative so that patients can embark on life-long multidisciplinary management of their condition and multisystem complications that continue to occur as they get older. Achieving an early diagnosis in practice requires a high index of suspicion for the condition and familiarity with the typical signs of TS to prompt genetic testing (5). In our cohort, the dominant referral triggers of short stature, antenatal findings, and menstrual disorders all fall within these consensus indications, but a third of diagnoses were made only in adolescence or adulthood, highlighting the value of broader awareness of these indications among non-specialist practitioners. In this regard, the recent clinical guidelines provide a useful set of indications to prompt testing (see **Table 3**).

**Table 3:** Indications for genetic testing to diagnose TS as recommended by the international turner syndrome consensus group (1, 5).

As the only clinical feature
Fetal cystic hygroma, or hydrops, especially when severe
Unexplained short stature
Left-sided outflow congenital heart defects (excluding BAV)
Unexplained delayed puberty/menarche, failure to progress puberty or secondary amenorrhea
Infertility
Characteristic physical features
At least two of the following
Renal anomaly (horseshoe, absence, or hypoplasia)
Madelung deformity
Neuropsychologic problems, and/or psychiatric issues
Multiple typical or melanocytic nevi
Dysplastic or hyperconvex nails
Other congenital heart defects (including BAV)
Hearing impairment <40 years of age together with short stature

There have been many studies of karyotype-phenotype associations in TS, but small sample sizes, heterogeneity of study design, uncertainty on the degree of mosaicism, and heterogeneous patient demographics mean that definitive karyotype-phenotype associations are still lacking (1, 2, 5, 12, 16, 17). The published data do, however, show that a specific karyotype does not always predict a specific phenotype, and new data on well annotated cohorts are always valuable to try to close this karyotype-phenotype gap. Our finding that age of menarche was significantly later and spontaneous menarche rare in individuals with monosomies compared with those with mosaicism or structural X-chromosome abnormalities is consistent with meta-analysis data of nearly 3000 females with TS, which reported spontaneous menarche in 20.8% (95% CI 19.3–22.4) of cases (compared with our 20%) and the lowest occurrence of spontaneous menarche in those with the 45, X karyotype (9.1%; 95% CI 7.3–11.3) (18). All patients, and especially those with 45, X monosomy, require careful monitoring of luteinizing hormone (LH), FSH, and anti-Müllerian hormone (AMH) from 8–9 years (for referral for fertility preservation as appropriate) and low-dose estrogen replacement between 11 and 12 years of age if FSH is elevated on at least two sequential measurements (1). Furthermore, knowledge of the karyotype helps practitioners counsel and manage expectations in patients and their families with respect to puberty, development, and likely future treatment needs.

We also found a higher frequency of autoimmune hypothyroidism in individuals with structural X-chromosome abnormalities (e.g., deletions, isochromosome Xq, and ring chromosomes) compared to those with monosomy or mosaicism. Previous studies have also reported an association between the isochromosome Xq karyotype and autoimmune hypothyroidism. Specifically, 83% of these patients tested positive for thyroid autoantibodies, and 37.5% exhibited autoimmune thyroid disease; rates significantly higher than those observed in patients with the 45, X karyotype (41% and 14%, respectively) (19). Similarly, Grossi et al. detected a higher prevalence of thyroid autoimmunity in isochromosome cases than in 45, X and mosaicism cases (20), while Al Alwan et al. detected autoimmune hypothyroidism in 18.8% of females with 45, X compared with 27.8% of females with other karyotypes, half with an isochromosome abnormality (21). Others have failed to detect this association (22). Similar data on associations between ring chromosomes and deletions are lacking. In our cohort, autoimmune hypothyroidism was present in 3/4 ring chromosome cases and 3/8 deletion cases; with such small subgroup sizes, the observations should be regarded as hypothesis-generating. For deletions, a meta-analysis showed that the site of deletion is likely to influence the thyroid disease phenotype (23).

Our finding of no detectable association between karyotype and GH therapy outcomes or responses is consistent with previous similar studies (12, 13, 24), although a recent small study detected poorer growth response outcomes for patients with monosomy or isochromosome abnormalities (14), especially during the second and third years of therapy. Overall, further evidence is needed to clarify karyotype-phenotype associations with GH responses, with the clinical imperative being to start therapy as early as possible to achieve optimal growth outcomes (1, 5).

We did not detect some other expected and established karyotype-phenotype associations (1, 5), such as between 45, X and more frequent comorbidities (25), although the evidence supporting this association is from studies of adult populations, the prevalence of related comorbidities increases with age, and the recording of comorbidities differs between studies. Our study was also limited by the relatively small cohort size, its retrospective nature with inherent risks of missing or incomplete data and information bias, and the possibility of inconsistent data recording between centers. Several variables that may influence outcomes were not consistently captured, including treatment adherence with GH therapy, the specific pubertal induction regimens used, nutritional factors, and detailed sub-classification of the ‘other’ congenital heart defects, which prevented us from incorporating CHD severity as a covariate in the growth response analyses. In addition, detailed molecular characterization (FISH, chromosomal microarray, or exome sequencing) was not performed, which may have missed low-level (≤10%) mosaicism not detectable by standard karyotyping. The karyotype-phenotype associations were primarily assessed using univariable tests, with multivariable models only fitted where the events-per-variable or observations-per-predictor exceeded the conventional minimum of 10 [(10); **Supplementary Table 5]**. In these analyses, the autoimmune hypothyroidism-karyotype association survived adjustment for current age, and the absence of a karyotype effect on the one-year GH response persisted after adjustment for GH dose, baseline height SDS, and age at initiation. The spontaneous menarche and three-year GH response analyses did not meet this threshold, so the univariable spontaneous menarche finding therefore could not be adjusted for baseline ovarian reserve or degree of mosaicism. Nevertheless, this multi-center study reflected real-world practice, supporting generalizability.

In conclusion, TS remains a challenging condition to recognize and manage, and all physicians must be aware of current management guidelines so that all patients receive multidisciplinary management as early as possible. While knowledge of karyotype-phenotype associations may not currently alter screening and management of girls and women with TS, it does help manage expectations and plan likely future management strategies, helping patients and their families adapt to their changing condition, life circumstances, and future medical events. This is especially true for likely management of puberty induction or vigilance for comorbidities, such as hypothyroidism. Individual and group trajectories showed the expected positive impact of GH therapy on growth, with no detectable difference in response between karyotype groups, and GH should be started as soon as the diagnosis is established in most cases.

## Data Availability

The original contributions presented in the study are included in the article/**Supplementary Material**. Further inquiries can be directed to the corresponding author.
