# Supplementary material for: Karyotype–phenotype associations in turner syndrome: a multicenter retrospective cohort study
Source: Front Endocrinol (Lausanne). 2026 Jul 7;17:1830126. doi: 10.3389/fendo.2026.1830126 (PMC13385663; doi:10.3389/fendo.2026.1830126)
Supplement: Supplementary file 1 [file Table1.docx]

**SUPPLEMENTARY MATERIAL**

**Supplementary Table S1.** Full list of karyotypes detected in the study population, reported using International System for Human Cytogenomic Nomenclature (ISCN) conventions. A small number of entries from the earliest records lack documented metaphase cell counts or carry lower-resolution band designations, reflecting variation in cytogenetic reporting over the study period.

| **Karyotype** | **n = 86** |
| --- | --- |
| 45,X | 38 (44.2%) |
| 45,X[11]/46,XX[41] | 4 (4.7%) |
| 45,X/46,X,del(X) | 1 (1.2%) |
| 45,X/46,XX | 2 (2.3%) |
| 45,X/46,XX,del(X)(q) | 1 (1.2%) |
| 45,X/46,XX/47,XXX | 1 (1.2%) |
| 45,X/46,XY | 1 (1.2%) |
| 45,X/47,XXX | 1 (1.2%) |
| 45,X/46,X,r(X)(p11.1q13.2) | 1 (1.2%) |
| 45,X[13]/46,X,idic(X)(p11.2)[23] | 1 (1.2%) |
| 45,X[19]/46,X,idic(X)(p11.2)[18] | 1 (1.2%) |
| 45,X[20]/46,X,i(X)(q10)[20] | 1 (1.2%) |
| 45,X[23]/46,X,r(X)(p22.3q28) | 1 (1.2%) |
| 45,X[2]/46,X,i(X)(q10)[28] | 1 (1.2%) |
| 45,X[30]/46,XY[20] | 1 (1.2%) |
| 45,X[31]/46,X,r(X)(p1q1)[29] | 2 (2.3%) |
| 45,X[37]/46,X,idic(X)(p11.2)[3] | 1 (1.2%) |
| 45,X[42]/47,XXX[8] | 1 (1.2%) |
| 45,X[4]/46,X,i(X)(q10)[46] | 1 (1.2%) |
| 45,X[4]/46,XX[65] | 1 (1.2%) |
| 45,X[5]/46,X,idic(X)(p11.2)[23] | 1 (1.2%) |
| 45,X[5]/46,XX[173] | 1 (1.2%) |
| 45,X[6]/46,XX[24] | 2 (2.3%) |
| 45,X[7]/46,X,idic(X)(q10)[43] | 1 (1.2%) |
| 45,X[8]/46,XX[20] | 1 (1.2%) |
| 45,X/46,X,i(X)(q10) | 1 (1.2%) |
| 46,X,del(X)(p11.2) | 1 (1.2%) |
| 46,X,del(X)(p21.1) | 1 (1.2%) |
| 46,X,del(X)(p22.3) | 1 (1.2%) |
| 46,X,del(X)(p22.3),del(X)(p11.2) | 1 (1.2%) |
| 46,X,del(X)(q21q27)[9]/46,XX[21] | 1 (1.2%) |
| 46,X,del(X)(q22) | 1 (1.2%) |
| 46,X,i(X)(q10) | 9 (10.5%) |
| 46,X,i(X)(q10)[20] | 1 (1.2%) |
| 46,X,idic(X)(p11.2) | 1 (1.2%) |

ISCN, International System for Human Cytogenomic Nomenclature; del, deletion; idic, isodicentric; i, isochromosome; r, ring chromosome.

**Supplementary Table S2.** One-way ANOVA (Welch's) of associations between continuous variables and karyotype group (monosomy, mosaicism, and structural X-chromosome abnormalities).

|  | **F statistic** | **p-value** |
| --- | --- | --- |
| **Age at diagnosis, years** | 1.758 | 0.187 |
| **FSH level, mIU/ml** | 0.664 | 0.524 |
| **Height SDS at diagnosis** | 0.211 | 0.811 |
| **% Change height SDS from baseline to 1 year** | 0.397 | 0.680 |
| **% Change height SDS from baseline to 3 years** | 0.597 | 0.560 |

**Supplementary Table S3.** Chi-squared tests of associations between categorical variables and karyotype group (monosomy, mosaicism, and structural X-chromosome abnormalities).

|  | **Monosomy (n=38)** | **Mosaicism (n=16)** | **Structural X-chromosome abnormalities (n=32)** | **Total (n=86)** | **p-value** |
| --- | --- | --- | --- | --- | --- |
| **Autoimmune hypothyroidism** |  |  |  |  | < 0.001^1^ |
| Missing | 1.0 | 0.0 | 0.0 | 1.0 |  |
| No | 32.0 (86.5%) | 15.0 (93.8%) | 17.0 (53.1%) | 64.0 (75.3%) |  |
| Yes | 5.0 (13.5%) | 1.0 (6.2%) | 15.0 (46.9%) | 21.0 (24.7%) |  |
| **Subclinical hypothyroidism** |  |  |  |  | 0.349^1^ |
| Missing | 1.0 | 0.0 | 0.0 | 1.0 |  |
| No | 33.0 (89.2%) | 16.0 (100.0%) | 28.0 (87.5%) | 77.0 (90.6%) |  |
| Yes | 4.0 (10.8%) | 0.0 (0.0%) | 4.0 (12.5%) | 8.0 (9.4%) |  |
| **Celiac disease** |  |  |  |  | 0.569^1^ |
| Missing | 11.0 | 3.0 | 8.0 | 22.0 |  |
| No | 23.0 (85.2%) | 12.0 (92.3%) | 19.0 (79.2%) | 54.0 (84.4%) |  |
| Yes | 4.0 (14.8%) | 1.0 (7.7%) | 5.0 (20.8%) | 10.0 (15.6%) |  |
| **Type 1 diabetes** |  |  |  |  | 0.109^1^ |
| No | 38.0 (100.0%) | 15.0 (93.8%) | 32.0 (100.0%) | 85.0 (98.8%) |  |
| Yes | 0.0 (0.0%) | 1.0 (6.2%) | 0.0 (0.0%) | 1.0 (1.2%) |  |
| **Type 2 diabetes** |  |  |  |  | 0.484^1^ |
| No | 37.0 (97.4%) | 15.0 (93.8%) | 29.0 (90.6%) | 81.0 (94.2%) |  |
| Yes | 1.0 (2.6%) | 1.0 (6.2%) | 3.0 (9.4%) | 5.0 (5.8%) |  |
| **Congenital heart disease** |  |  |  |  | 0.193^1^ |
| No | 18.0 (47.4%) | 11.0 (68.8%) | 21.0 (65.6%) | 50.0 (58.1%) |  |
| Yes | 20.0 (52.6%) | 5.0 (31.2%) | 11.0 (34.4%) | 36.0 (41.9%) |  |
| **Renal anomalies** |  |  |  |  | 0.490^1^ |
| Missing | 3.0 | 1.0 | 2.0 | 6.0 |  |
| No | 27.0 (77.1%) | 11.0 (73.3%) | 26.0 (86.7%) | 64.0 (80.0%) |  |
| Yes | 8.0 (22.9%) | 4.0 (26.7%) | 4.0 (13.3%) | 16.0 (20.0%) |  |
| **Hypertension** |  |  |  |  | 0.411^1^ |
| No | 34.0 (89.5%) | 13.0 (81.2%) | 30.0 (93.8%) | 77.0 (89.5%) |  |
| Yes | 4.0 (10.5%) | 3.0 (18.8%) | 2.0 (6.2%) | 9.0 (10.5%) |  |
| **Dyslipidemia** |  |  |  |  | 0.503^1^ |
| Missing | 14.0 | 4.0 | 14.0 | 32.0 |  |
| No | 18.0 (75.0%) | 7.0 (58.3%) | 11.0 (61.1%) | 36.0 (66.7%) |  |
| Yes | 6.0 (25.0%) | 5.0 (41.7%) | 7.0 (38.9%) | 18.0 (33.3%) |  |
| **Conductive hearing loss** |  |  |  |  | 0.858^1^ |
| No | 31.0 (81.6%) | 14.0 (87.5%) | 27.0 (84.4%) | 72.0 (83.7%) |  |
| Yes | 7.0 (18.4%) | 2.0 (12.5%) | 5.0 (15.6%) | 14.0 (16.3%) |  |
| **Sensorineural hearing loss** |  |  |  |  | 0.354^1^ |
| No | 38.0 (100.0%) | 15.0 (93.8%) | 31.0 (96.9%) | 84.0 (97.7%) |  |
| Yes | 0.0 (0.0%) | 1.0 (6.2%) | 1.0 (3.1%) | 2.0 (2.3%) |  |
| **Recurrent otitis media** |  |  |  |  | 0.812^1^ |
| No | 28.0 (73.7%) | 13.0 (81.2%) | 25.0 (78.1%) | 66.0 (76.7%) |  |
| Yes | 10.0 (26.3%) | 3.0 (18.8%) | 7.0 (21.9%) | 20.0 (23.3%) |  |
| **Musculoskeletal abnormalities** |  |  |  |  | 0.535^1^ |
| No | 28.0 (73.7%) | 14.0 (87.5%) | 25.0 (78.1%) | 67.0 (77.9%) |  |
| Yes | 10.0 (26.3%) | 2.0 (12.5%) | 7.0 (21.9%) | 19.0 (22.1%) |  |
| **Skin disorder** |  |  |  |  | 0.480^1^ |
| No | 29.0 (76.3%) | 14.0 (87.5%) | 23.0 (71.9%) | 66.0 (76.7%) |  |
| Yes | 9.0 (23.7%) | 2.0 (12.5%) | 9.0 (28.1%) | 20.0 (23.3%) |  |
| **Eye disorder** |  |  |  |  | 0.923^1^ |
| No | 29.0 (76.3%) | 13.0 (81.2%) | 25.0 (78.1%) | 67.0 (77.9%) |  |
| Yes | 9.0 (23.7%) | 3.0 (18.8%) | 7.0 (21.9%) | 19.0 (22.1%) |  |

^1^ Pearson chi-squared test.

**Supplementary Table S4.** Subgroup analysis of associations between karyotypes and autoimmune hypothyroidism.

|  | | **Autoimmune hypothyroidism** | |  |
| --- | --- | --- | --- | --- |
| **Karyotype subtype** |  | **No** | **Yes** | **Total** |
| Deletion | Observed | 5 | 3 | 8 |
|  | % within row | 62.5% | 37.5% | 100.0% |
| Isochromosome | Observed | 11 | 9 | 20 |
|  | % within row | 55.0% | 45.0% | 100.0% |
| Monosomy | Observed | 32 | 5 | 37 |
|  | % within row | 86.5% | 13.5% | 100.0% |
| Mosaicism with triple x | Observed | 3 | 0 | 3 |
|  | % within row | 100.0% | 0.0% | 100.0% |
| Mosaicism | Observed | 10 | 1 | 11 |
|  | % within row | 90.9% | 9.1% | 100.0% |
| Ring chromosome | Observed | 1 | 3 | 4 |
|  | % within row | 25.0% | 75.0% | 100.0% |
| Y mosaicism | Observed | 2 | 0 | 2 |
|  | % within row | 100.0% | 0.0% | 100.0% |
| Total | Observed | 64 | 21 | 85 |
|  | % within row | 75.3% | 24.7% | 100.0% |

**Supplementary Table S5.** Multivariable models for karyotype-phenotype associations meeting the pre-specified events-per-variable (or observations-per-predictor) threshold of ≥10.

**Panel A. Autoimmune hypothyroidism (multivariable logistic regression; n=85, 21 events).**

| **Variable** | **Adjusted OR** | **95% CI** | **p-value** |
| --- | --- | --- | --- |
| Karyotype: Monosomy (reference) | 1.00 | — | — |
| Karyotype: Mosaicism | 0.24 | 0.02–2.65 | 0.243 |
| Karyotype: Structural X-chromosome abnormality | 5.22 | 1.55–17.53 | 0.008 |
| Current age (per year) | 1.07 | 1.01–1.14 | 0.025 |

*Sensitivity analysis (binary karyotype contrast; events-per-variable 10.5):*

| **Variable** | **Adjusted OR** | **95% CI** | **p-value** |
| --- | --- | --- | --- |
| Structural X-chromosome abnormality (vs not) | 7.29 | 2.32–22.97 | 0.001 |
| Current age (per year) | 1.06 | 1.00–1.12 | 0.036 |

**Panel B. Percentage change in height standard deviation score at 1 year of GH treatment (multivariable linear regression; n=58).**

| **Variable** | **β (% change)** | **95% CI** | **p-value** |
| --- | --- | --- | --- |
| Karyotype: Monosomy (reference) | 0 | — | — |
| Karyotype: Mosaicism | +21.87 | −0.15 to +43.89 | 0.052 |
| Karyotype: Structural X-chromosome abnormality | +0.36 | −13.98 to +14.71 | 0.960 |
| GH dose (per mg/kg) | +664.12 | −42.51 to +1370.75 | 0.065 |
| Baseline height SDS (per SDS) | +6.27 | −2.16 to +14.70 | 0.142 |
| Age at GH initiation (per year) | −0.44 | −2.13 to +1.26 | 0.606 |

Overall model R^2^ = 0.161, adjusted R^2^ = 0.080, F p = 0.096.

Threshold for inclusion of an analysis: events-per-variable ≥10 for logistic regression and observations-per-predictor ≥10 for linear regression. The spontaneous menarche outcome (17 events) and three-year GH response (observations-per-predictor 9.6) did not meet this threshold and are reported as univariable analyses only (Supplementary Tables S2–S3).
